# B-cells in breast cancer: current insights and challenges

**DOI:** 10.3389/fonc.2026.1883381

**Published:** 2026-08-07

**Authors:** Bo Xiang, Tao Liu, Duo Xu, Wei Wang

**Affiliations:** Department of Breast Surgery, Jilin Cancer Hospital, Changchun, China

**Keywords:** B cells, breast cancer, immunotherapy, tertiary lymphoid structures, tumor microenvironment

## Abstract

The clinical success of immune checkpoint blockade and adoptive T-cell therapies has cemented a T-cell-centric paradigm in cancer immunology, inadvertently overshadowing the humoral arm of adaptive immunity. However, mounting evidence now demands a recalibration of this view, particularly in breast cancer, where tumor-infiltrating B cells (TIL-Bs), including terminally differentiated plasma cells, and the antibodies they produce emerge as active architects of the tumor microenvironment rather than passive bystanders. In this Review, we synthesize current insights into B-cell biology across the breast cancer ecosystem, from developmental ontogeny to functional specialization within tertiary lymphoid structures (TLSs). We dissect the dualistic nature of TIL-Bs: on one hand, clonally expanded, class-switched plasma cells and TLS-associated germinal center reactions drive potent anti-tumor immunity through antibody-dependent cytotoxicity, antigen presentation, and T-cell collaboration; on the other hand, regulatory B cells, atypical memory subsets, and pathogenic antibody isotypes foster immunosuppression, angiogenesis, and metastatic dissemination. We further examine the prognostic and predictive value of B-cell metrics across molecular subtypes, highlighting their association with response to neoadjuvant chemotherapy, anti-HER2 therapy, and immune checkpoint inhibition, especially in triple-negative and HER2-positive disease. Finally, we discuss emerging therapeutic strategies to harness anti-tumor B-cell responses while addressing the significant challenges of functional stratification, biomarker standardization, and the translation of preclinical insights into clinical benefit. Integrating B-cell function, rather than mere quantity, into next-generation pathological reporting and adaptive trial designs represents an essential frontier for precision oncology in breast cancer.

## Introduction

The advent of cancer immunotherapy has fundamentally reshaped the therapeutic landscape for solid malignancies (1). Historically, the conceptual framework of tumor immunology has been heavily dominated by cellular immunity. The remarkable clinical efficacy of immune checkpoint blockade (ICB) and adoptive cell therapies solidified the consensus that CD8^+^ cytotoxic T cells are the principal effectors of tumor regression (2, 3). Consequently, the tumor microenvironment (TME) has been interrogated largely through a T-cell-centric lens, with CD8^+^ cytotoxic T cells heralded as the cardinal effectors of tumor regression and the principal biomarkers of immunotherapy response (4–7). While this approach has yielded transformative clinical benefits, it has left the humoral arm of adaptive immunity comparatively under-explored, often relegating B lymphocytes to the periphery of immuno-oncology research.

Mounting evidence now demands a recalibration of this view. B cells, including their terminally differentiated plasma cell (PC) counterparts, and the antibodies they produce are not passive bystanders in the TME but active participants that can profoundly influence tumor control (8–11). Through the production of tumor-specific antibodies, antigen presentation to T cells, and the organization of tertiary lymphoid structures (TLSs), B cells can drive potent anti-tumor immunity (12, 13). Conversely, distinct B-cell subsets, including regulatory B cells (Bregs) and certain immunoglobulin A (IgA)-secreting plasma cells, which can function as regulatory cells by expressing immunosuppressive molecules such as IL-10 and PD-L1, can foster an immunosuppressive milieu, promoting tumor survival and immune evasion (14–18). The functional outcome of B-cell infiltration is thus context-dependent, dictated by the differentiation state of the cells, the anatomical maturity of TLSs, and the isotype and specificity of the antibodies produced (19, 20).

Breast cancer, the most frequently diagnosed malignancy among women worldwide, exemplifies both the promise and the complexity of incorporating B-cell biology into clinical oncology (21). Long considered immunologically heterogeneous compared with melanoma or non-small cell lung cancer, breast cancer is now recognized to harbor substantial immune infiltrates, particularly in triple-negative (TNBC) and HER2-enriched subtypes (22–24). Tumor-infiltrating lymphocytes (TILs) in these aggressive subtypes are established prognostic and predictive biomarkers, yet the vast majority of clinical and translational research has focused on T-cell subsets (25, 26). Tumor-infiltrating B cells (TIL-Bs) constitute approximately 20% of the immune infiltrate in breast carcinoma and display remarkable phenotypic diversity, spanning naive B cells, memory subsets, GC B cells, and terminally differentiated plasma cells (27–29). Retrospective clinical studies have linked higher TIL-Bs densities to improved survival and enhanced response to neoadjuvant chemotherapy, particularly in TNBC (21, 30, 31). However, these associations are not universal. In certain cohorts, adverse outcomes have been correlated with the presence of CD138^+^ plasma cells, which may drive Immunosuppression (32–34). Similarly, Bregs can directly suppress cytotoxic T-cell responses via IL-10 and TGF-β secretion, leading to poor prognosis (35–38).

While several recent reviews have addressed the broad prognostic significance of tumor-infiltrating lymphocytes or cataloged the general presence of B cells in solid tumors (10, 39, 40), this Review uniquely distinguishes itself by focusing on the functional and spatial qualitative heterogeneity of the B-cell compartment within the breast cancer ecosystem. Rather than treating B cells as a uniform population, we synthesize recent high-resolution single-cell and spatial transcriptomic insights to dissect the delicate balance between anti-tumor effector states and highly specialized immunosuppressive or pro-metastatic subsets, such as regulatory B cells (Bregs) and atypical memory (AtM) B cells.

**Figure 1:**
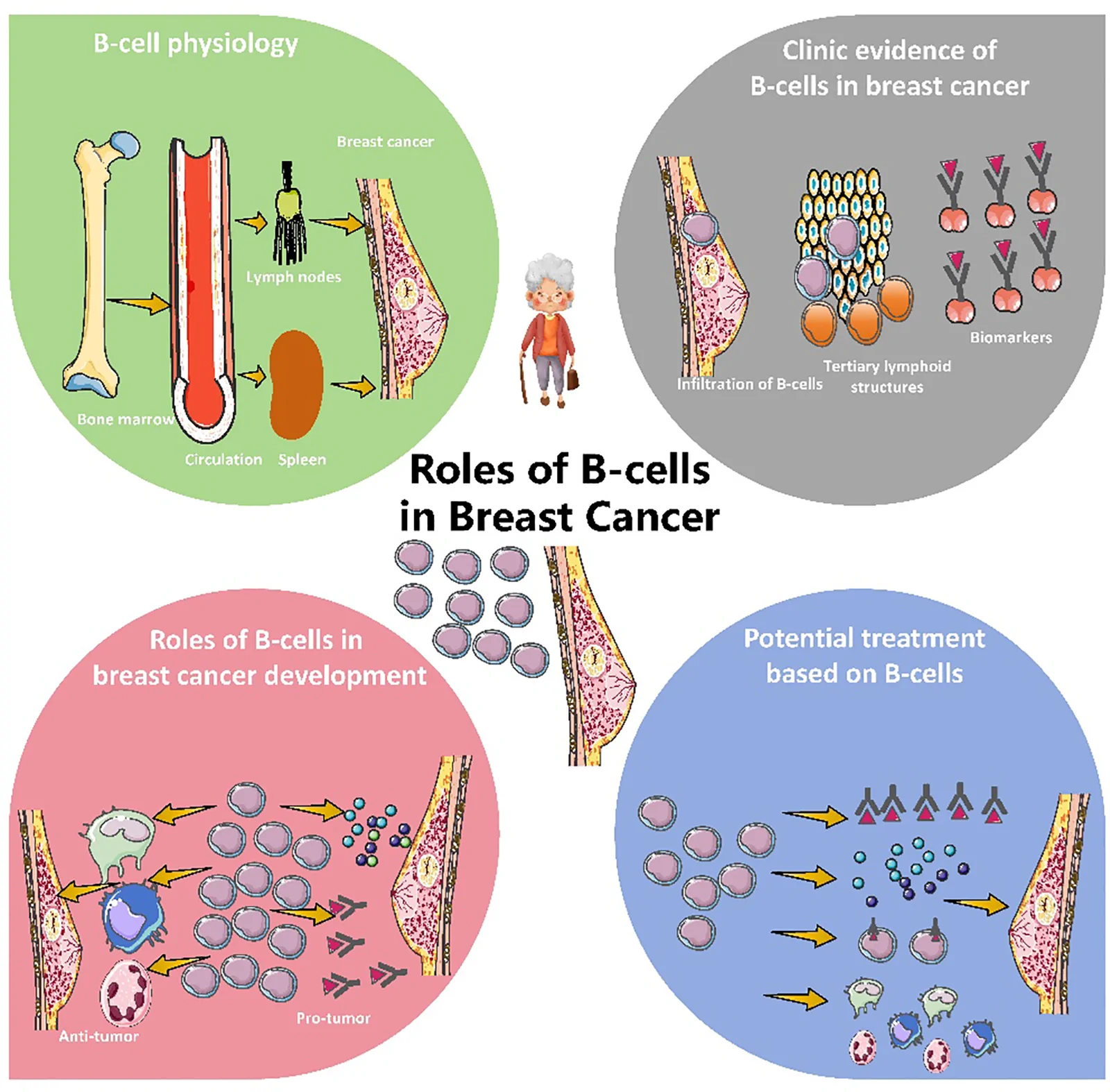
Schematic overview of the review structure. This image outlines the four core themes discussed: (1) B-cell physiology, tracing development from the bone marrow to the periphery; (2) Clinical evidence, highlighting the prognostic value of tumor-infiltrating B-cells and tertiary lymphoid structures (TLSs); (3) Roles in breast cancer, dissecting the dualistic anti-tumor and pro-tumor functions of B-cells; and (4) Potential treatments, exploring emerging strategies to therapeutically target or reprogram B-cell immunity.

In this Review, we synthesize the current understanding of B-cell biology within the breast cancer ecosystem, traversing from fundamental B-cell physiology to the intricate cellular and molecular interactions that govern their tumor-modulating functions (**Figure 1**). We examine the clinical evidence linking TIL-Bs, particularly effector subsets such as plasma cells and their spatial organization within TLSs, to patient prognosis and treatment response across breast cancer subtypes. We dissect the mechanisms by which B cells can either restrain or promote tumor progression, including their roles in antigen presentation, cytokine secretion, and antibody-mediated immune modulation. Finally, we explore how these insights are paving the way for novel therapeutic interventions. By confronting current hurdles, such as subset-specific targeting and the need for rigorous diagnostic biomarker frameworks, we emphasize how expanding our focus to include the full spectrum of adaptive immunity can unlock new vulnerabilities in breast cancer and ultimately refine precision oncology.

## B-cell physiology

### Developmental ontogeny and B-cell receptor diversification

During adult life in mammals, B cells arise from hematopoietic stem cells in the bone marrow, where they progress through a tightly regulated developmental program marked by sequential immunoglobulin gene rearrangement (41–43). During the pro-B and pre-B stages, V(D)J recombination assembles the heavy and light chains that collectively generate the B-cell receptor (BCR), a membrane-bound antibody complex comprising two identical heavy chains and two light chains (44, 45). Successful rearrangement yields immature B cells expressing surface IgM and IgD (44–46). Before egress into the periphery, these cells undergo stringent negative selection through clonal deletion, receptor editing, and anergy to enforce central tolerance and purge autoreactive specificities (47, 48).

### Peripheral maturation and antigen-driven activation

Upon entering the bloodstream and secondary lymphoid organs (SLOs), immature B cells mature into naive follicular B cells (IgM^+^IgD^+^CD19^+^CD20^+^CD21^+^CD40^+^), poised to encounter cognate antigen (49). Antigen engagement triggers clonal expansion and initiates two principal differentiation trajectories (49, 50). In the extrafollicular pathway, activated B cells rapidly differentiate into short-lived plasmablasts that secrete low-affinity, largely unmutated antibodies (51, 52). Alternatively, B cells may enter a germinal center (GC) reaction, a transient microanatomical structure wherein iterative cycles of somatic hypermutation and affinity-based selection occur (53, 54). Within the GC dark zone, B cells proliferate and accumulate point mutations in their BCR variable-region genes; they then migrate to the light zone, where follicular dendritic cells and T follicular helper (Tfh) cells mediate selection of high-affinity variants (55–57). The outputs of productive GC reactions are memory B cells and long-lived plasma cells (PCs), the latter representing the terminally differentiated, antibody-secreting effectors of the lineage (54, 58, 59).

### Functional subsets and phenotypic specialization

After emigrating from the bone marrow, B cells reach a mature state that encompasses both antigen-inexperienced naive populations and antigen-experienced memory subsets. Conventional memory B cells (classically CD19+ CD20+ CD27+ IgD- in humans) carry somatically mutated, class-switched BCRs and can rapidly re-enter GCs or differentiate into antibody-secreting cells upon rechallenge (60, 61). It is critical to recognize species-specific differences when categorizing innate-like B-cell populations. In mice, B-1 cells represent a well-defined, distinct lineage that responds rapidly to T-independent antigens (62). In humans, however, the functional equivalents of murine B-1 cells are primarily considered to be IgM+ memory B cells and Marginal Zone (MZ) B cells, which mediate similar rapid innate-like humoral responses (63). Conversely, follicular B cells are conserved across species and orchestrate classical T-dependent humoral responses.

Regulatory B cells (Bregs) are identified primarily by their secretion of IL-10, IL-35, and TGF-beta rather than by a uniform surface signature (64–67). These cells suppress effector T-cell responses, promote regulatory T-cell (Treg) expansion, and maintain immune tolerance (64, 66, 68–71). More recently, atypical memory (AtM) B cells, also termed age-associated B cells or CD27- IgD- double-negative cells, have been characterized by the expression of CD11c and the transcription factor ZEB2 (72–77). Emerging under chronic antigenic stimulation, these cells were previously considered terminally exhausted. However, this view is overly simplistic, as AtM B cells retain the capacity to differentiate into plasma cells under appropriate stimulatory conditions, alongside their potential to secrete autoreactive antibodies that dysregulate immune homeostasis (72, 73, 78).

### Antibody isotypes and effector potential

A critical determinant of humoral immunity is the antibody class generated through class-switch recombination (CSR) (79, 80). Naive B cells express IgM; upon activation, they may switch to IgG, IgA, or IgE. IgG1 and IgG3 subclasses engage Fcγ receptors on myeloid and natural killer (NK) cells to mediate antibody-dependent cellular cytotoxicity (ADCC), antibody-dependent cellular phagocytosis (ADCP), and complement activation, mechanisms integral to anti-tumor defense (81–84). In contrast, IgG2 and IgG4 are generally associated with less favorable inflammatory outcomes, while IgA, abundant at mucosal sites, can form immune complexes that favor suppressive myeloid phenotypes (47, 49, 85). Long-lived PCs, which can survive for decades and serve as the principal source of these antibodies, predominantly reside within specialized survival niches in the bone marrow, although they can also be found in peripheral tissues where they sustain local humoral immunity (86, 87).

## Clinical evidence of B-cells in breast cancer

Retrospective analyses of archival tumor specimens and correlative studies embedded within prospective clinical trials have established that the presence, density, and phenotypic composition of tumor-infiltrating B cells (TIL-Bs) carry independent prognostic and predictive information in breast cancer (31, 88). Although initial observational studies relied primarily on immunohistochemical detection of pan–B-cell markers such as CD20, more recent multiplex imaging, bulk transcriptomic deconvolution, and single-cell atlases have refined these associations by linking specific B-cell states and spatial architectures to patient outcomes across molecular subtypes (**Table 1**) (89, 90).

**Table 1:** Clinical significance of B-cell-related biomarkers in breast cancer.

Biomarker	Breast cancer subtype	Findings	Reference
Intratumoral B-cell abundance	TNBC, HER2-positive	Abundant tumor-infiltrating B cells correlated directly with longer disease-free survival (DFS) and overall survival (OS).	(13)
IgG gene-expression signatures, CD20+ cell counts	HER2-positive	High baseline IgG signatures and TILs were associated with increased pCR rates	(113)
Baseline B-cell abundance and TLS maturity	TNBC	Strongly linked to a favorable response to neoadjuvant pembrolizumab combined with chemotherapy.	(120)
Active germinal centers (GCs) in tumor-draining lymph nodes	TNBC	Nodal GC presence is associated with a significantly lower risk of distant metastasis	(102)
Baseline CD20+ B cells, CD38+ plasma cells, and TLS density	TNBC	High baseline densities were independently associated with higher pCR rates	(109)
B-cell abundance in the periductal stroma	HER2-positive DCIS	Inversely correlated with epithelial to mesenchymal transition programs	(127)

### Prognostic value of B-cell infiltration and subsets

In invasive breast carcinoma, higher densities of CD20^+^ TIL-Bs have been consistently associated with improved disease-free survival (DFS) and overall survival (OS), particularly in triple-negative breast cancer (TNBC) and HER2-positive disease (90–92). In the BIG 02–98 adjuvant trial cohort, abundant tumor-infiltrating B cells correlated with longer DFS and OS in both HER2-positive and TNBC patients, and B cells isolated from these tumors expressed a broad repertoire of pro-inflammatory cytokines (13). Similarly, a large observational study of patients with operable invasive ductal carcinoma reported that elevated plasma cell infiltration was an independent favorable prognostic factor (93). These findings are reinforced by single-cell and B-cell receptor (BCR) repertoire analyses in TNBC, where memory B cells are significantly enriched relative to normal breast tissue and are linked to better clinical outcomes (94–96).

Nevertheless, the relationship is not uniformly favorable. The functional heterogeneity of B-cell subsets means that prognostic signals are context-dependent. In TNBC, an IgG-biased, clonally expanded B-cell phenotype identified by high-dimensional BCR profiling is associated with superior survival, whereas the presence of IL-10-producing regulatory B cells (Bregs) correlates with increased regulatory T-cell infiltration and shorter relapse-free intervals (97, 98). Likewise, high densities of CD138^+^ plasma cells have been identified as an independent favorable factor for progression-free and OS in some TNBC cohorts, yet tumor-specific IgG autoantibody signatures have also been linked to paradoxically shorter DFS in certain patient populations, underscoring the complexity of humoral immunity in breast cancer (34, 35). Spatial context further modulates these associations: dispersed stromal infiltration of B cells along the tumor border is associated with a lower incidence of recurrence, whereas the physical proximity of B-cell aggregates to malignant cell islands appears to influence immune-mediated killing capacity (21, 99).

### Tertiary lymphoid structures and nodal immunity

Beyond simple cell density, the organizational pattern of B-cell infiltration is clinically meaningful. Tertiary lymphoid structures (TLSs), ectopic lymphoid aggregates containing CD20^+^ B-cell zones, CD3^+^ T-cell areas, and mature dendritic cells, are detected in TNBC specimens and are strongly associated with improved DFS and OS across breast cancer subtypes (30). A 12-chemokine gene signature predictive of TLS presence has been shown to stratify untreated breast cancer patients by survival, while histologic detection of TLSs confers favorable prognosis independent of conventional stage (8, 21, 30, 99). The maturational stage of these structures dictates their clinical impact. Mature TLSs harboring active germinal-center reactions are linked to productive anti-tumor immunity, whereas immature structures correlate with immune evasion driven by metabolic and transcriptional dysfunctions (100, 101).

Systemic lymphoid tissue also provides prognostic data. In TNBC patients with axillary nodal involvement, the presence of active germinal centers (GCs) in tumor-draining lymph nodes is associated with a significantly lower risk of distant metastasis, even among patients with a low overall tumor-infiltrating lymphocyte (TIL) density at the primary site (102). Deep-learning quantification of GC frequency in cancer-free and involved nodes has validated this association, suggesting that nodal B-cell activation reflects a systemic anti-tumor immune capacity that complements the local microenvironment (103–106).

### Predictive biomarkers for therapy response

B-cell metrics have emerged as predictive biomarkers for neoadjuvant chemotherapy (NAC), immunotherapy, and anti-HER2 regimens (107, 108). In TNBC, high baseline densities of CD20^+^ B cells, CD38^+^ plasma cells, and TLSs have been independently associated with higher pathologic complete response (pCR) rates following anthracycline- and taxane-based NAC (109). Automated quantitative analysis of intratumoral CD20 expression predicted pCR with high odds ratios in multi-agent neoadjuvant cohorts, and tumors with elevated plasma-cell infiltration showed significantly improved response to sequential chemotherapy (110, 111). Notably, chemotherapy itself dynamically reshapes the B-cell compartment. As detailed in subsequent sections, treatment induces specific phenotypic shifts that correlate directly with therapeutic efficacy and long-term survival (112).

In HER2-positive disease, correlative analyses from the CALGB 40601 and TBCRC006 trials demonstrated that high immunoglobulin G (IgG) gene-expression signatures and elevated intratumoral CD20^+^ cell counts were associated with increased pCR rates following neoadjuvant anti-HER2 therapy, with or without chemotherapy (113–116). These observations align with the concept that pre-existing humoral immunity augments antibody-dependent cellular cytotoxicity (ADCC) and antigen-presenting functions that synergize with trastuzumab and pertuzumab (89, 117, 118).

With the integration of immune checkpoint inhibitors (ICIs) into standard care, B-cell parameters are being evaluated as predictive biomarkers for immunotherapy benefit. In early-stage TNBC treated with pembrolizumab plus chemotherapy, baseline B-cell abundance and TLS maturity have been linked to favorable response (119, 120). A nine-gene B-cell cytokine signature derived from pretreatment TNBC specimens was shown to predict response to anti–PD-1/anti–CTLA-4 blockade and to stratify OS in validation cohorts spanning lung, colorectal, and melanoma cancers, supporting the translatability of B-cell–centric predictive signatures (121). More recently, integrative single-cell and spatial transcriptomic analyses of breast cancer samples have identified CD200^+^ naive B cells and ISG15^+^ atypical memory B cells as tumor-enriched populations; the former are tightly associated with TLS formation, improved clinical outcomes, and enhanced anti–PD-1 efficacy, whereas proliferative CD19^+^Ki67^+^ B-cell subsets have been correlated with poor OS and increased brain metastasis risk in TNBC (122, 123).

### Systemic and dynamic B-cell measures

The clinical relevance of B cells extends beyond the primary tumor. Peripheral blood analyses reveal that dynamic changes in circulating B-cell percentages during systemic therapy may mirror treatment response (124, 125). In a prospective observational cohort, breast cancer patients who exhibited decreased peripheral B-cell frequencies paired with increased natural killer cell percentages after subtype-guided therapy tended to achieve higher objective response rates (124). Conversely, systemic immune remodeling in advanced disease includes marked peripheral lymphopenia with a selective enrichment of TNFR2-expressing lymphocyte subsets, including both B cells and CD3^+^ T cells, which suggests that these circulating immune phenotypes could serve as minimally invasive biomarkers for disease stage and therapeutic vulnerability (126).

### Emerging spatial and multi-omic clinical atlases

High-resolution spatial transcriptomic and single-cell profiling of human breast cancer specimens are now refining how B-cell evidence is translated into clinical practice. A multi-omic study of HER2-positive ductal carcinoma *in situ* (DCIS) using spatial transcriptomics found that B-cell abundance in the periductal stroma was inversely correlated with epithelial–mesenchymal transition programs and positively associated with metabolic gene expression, implying a protective role against invasive progression (127). Similarly, multi-omics integration in occult breast cancer (OBC) has demonstrated that B-cell–mediated immune surveillance in axillary lymph nodes is associated with primary tumor clearance and favorable survival trajectories relative to non-occult stage-matched controls (128). These data support the incorporation of B-cell spatial metrics and TLS assessment into next-generation pathologic reporting frameworks (127, 129).

## Roles of B-cells in breast cancer development

The functional impact of B cells on breast cancer biology is governed by a spectrum of cellular programs that can either constrain or facilitate tumor progression (21). These opposing roles are not mutually exclusive; rather, they reflect the phenotypic plasticity of B-cell subsets, the maturational state of the humoral response, the isotype and specificity of antibodies produced, and the dynamic cues present within the tumor microenvironment (TME) (130). A comprehensive mechanistic understanding of these divergent functions is essential for rational therapeutic targeting (**Table 2**).

**Table 2:** B-cell biomarkers and their clinical outcomes.

B-cell subset	Markers	Primary functions	Prognostic association
Naive B cells	IgM+, IgD+, CD19+, CD20+, CD21+, CD40+, CD200+	Poised to encounter cognate antigen; CD200+ subsets are tightly associated with TLS formation	Generally favorable; linked to improved clinical outcomes and enhanced anti-PD-1 efficacy.
Memory B cells	CD19+, CD20+, CD27+, IgD-	Rapidly re-enter germinal centers or differentiate into antibody-secreting cells upon rechallenge	Favorable; significantly enriched in TNBC and linked to better clinical outcomes
Plasma cells (IgG+)	CD138+, CD38+, intracellular IgG	Terminally differentiated effector cells; secrete high-affinity tumor-specific antibodies to drive ADCC, ADCP, and complement activation	Favorable; independent factor for improved progression-free and overall survival
Plasma cells (IgA+/IgG4+)	CD138+, CD38+, intracellular IgA or IgG4	Form immune complexes that favor suppressive myeloid phenotypes; induce tolerogenic M2 macrophage activation	Unfavorable; associated with immune evasion and suppression of cytotoxic T-lymphocyte expansion
Regulatory B cells (Bregs)	IL-10+, TGF-beta+, IL-35+ (lack univocal surface markers)	Suppress effector T-cell responses, promote regulatory T-cell (Treg) expansion, and maintain immune tolerance	Unfavorable; correlates directly with Treg infiltration and inversely with relapse-free survival
Atypical memory (AtM) B cells	CD27-, IgD-, CD11c+, ZEB2+, ISG15+	Display an exhausted phenotype, secrete autoreactive antibodies, and express elevated PD-L1 to maintain an immunosuppressive milieu	Unfavorable; associated with chronic antigenic stimulation and immune evasion
Proliferative B cells	CD19+, Ki67+	Enhance tumor organoid proliferation, invasion, and metastatic potential through NAMPT/ITGA5/ITGB1 signaling	Unfavorable; correlated with poor overall survival and increased brain metastasis risk in TNBC.

### Anti-tumor effector functions: antibodies, antigen presentation, and direct cytotoxicity

B cells contribute to anti-tumor immunity through multiple complementary pathways. Following productive germinal center (GC) reactions, tumor-specific antibodies of the IgG1 and IgG3 isotypes opsonize malignant cells. As introduced previously, these isotypes execute potent anti-tumor defense by triggering ADCC, ADCP, and the classical complement cascade (43, 89, 131). In breast cancer, tumor-infiltrating plasma cells exhibit evidence of extensive somatic hypermutation and clonal expansion, with IgG isotype switching correlating with improved prognosis (98, 132, 133). Emerging data from single-cell atlases indicate that plasma cells in certain TNBC cohorts display the highest frequency of IgH somatic hypermutation among all TIL-B subsets, which points toward potentially robust *in situ* antigen-driven selection against tumor-associated antigens (134).

Beyond humoral output, B cells function as professional antigen-presenting cells (APCs) (135). By internalizing tumor-derived antigens via the B-cell receptor and presenting peptides via MHC class II, they activate CD4^+^ T cells; through co-stimulatory ligands such as CD80/CD86 and the CD40/CD40L axis, they potentiate cytotoxic CD8^+^ T-cell responses (136). Intratumoral B–T crosstalk induces the differentiation of tumor-specific follicular helper T (Tfh) cells, which secrete IL-21 to amplify CD8^+^ T-cell cytotoxicity (137, 138). Effector B cells further secrete chemokines that recruit T cells into the TME, and memory B cells produce CXCL8 to attract dendritic cells to metastatic sites, reinforcing antigen presentation (139). Notably, subsets of granzyme B-positive (GzmB^+^) or Fas ligand–expressing (FasL^+^) B cells can mediate direct cytotoxicity against breast cancer cells through the Fas–FasL and perforin–granzyme pathways (140–142).

### Immunosuppressive and pro-tumorigenic programs: Bregs, pathogenic antibodies, and dysfunctional states

Conversely, distinct B-cell states actively promote tumor progression and immune evasion. Regulatory B cells (Bregs) infiltrating breast tumors secrete IL-10, TGF-β, and IL-35, which suppress macrophage and NK-cell cytotoxicity, inhibit Th1 and Th17 differentiation, and drive the conversion of naive CD4^+^ T cells into regulatory T cells (Tregs) (143–145). In TNBC, the abundance of IL-10^+^ Bregs correlates directly with Treg infiltration and inversely with relapse-free survival (146). Atypical memory (AtM) B cells, characterized by CD11c and ZEB2 expression, display an exhausted phenotype, secrete autoreactive antibodies, and express elevated PD-L1, thereby maintaining an immunosuppressive TME (147). In breast cancer, stress-response memory B cells highly expressing stress-related genes are associated with poor prognosis and non-response to immunotherapy (148).

The antibody isotype profoundly influences tumor biology. In contrast to the robust inflammatory functions of IgG1 and IgG3, locally produced IgA and IgG4 lack these effector capacities and instead favor immune tolerance (149, 150). IgA^+^ plasma cells can inhibit cytotoxic T-lymphocyte expansion through IL-10, PD-L1, and FasL, and IgA immune complexes can polarize macrophages toward an M2-like phenotype (151–153). In breast cancer, preclinical murine models suggest that tumor-derived IgG targeting HSPA4 may activate the Src/NF-κB pathway and facilitate lymph node metastasis via the CXCR4/SDF-1α axis (154). Similarly, studies in other solid malignancies indicate that IgG4 subclass antibodies induce tolerogenic M2 macrophage activation and are associated with tumor malignancy. These findings illustrate that the humoral response might be redirected to serve pro-tumorigenic ends depending on the dominant isotype, a concept that is actively being explored as an analogy in breast cancer (155, 156).

### Modulation of tumor proliferation, metastasis, and the microenvironment

Experimental models suggest that B cells might also actively reshape the TME beyond immune modulation. In these models, tumor-associated B cells can promote angiogenesis by secreting vascular endothelial growth factor (VEGF), CYR61, ADM, FGF2, PDGFA, and MDK (43). A pro-angiogenic CD49b^+^CD73^+^ B-cell subset has been identified in non-breast cancer contexts, and STAT3-activated B cells in melanoma enhance tumor progression through angiogenesis. This suggests that analogous STAT3 signaling pathways in B cells might represent a therapeutic target in breast cancer, although direct validation is still required (157, 158). B cells can also indirectly stimulate angiogenesis by secreting IgG and polarizing macrophages toward pro-tumorigenic phenotypes (42, 52).

With respect to metastatic dissemination, recent mechanistic studies utilizing *in vitro* assays and animal models have proposed that cycling CD19^+^Ki67^+^ B cells can enhance TNBC organoid proliferation, invasion, and brain metastatic potential through activation of the NAMPT/ITGA5/ITGB1 signaling axis (122). In bone metastasis models, distinct developmental stages of bone marrow B cells exert opposing effects on tumor progression: CD20^-^ pro/pre-B cells promote tumor cell proliferation and stem-like properties through the secretion of soluble factors, whereas CD20^+^ immature B cells restrict intraosseous tumor growth and are clinically correlated with prolonged overall survival (159). Conversely, osteoprotegerin (OPG)-producing CD19^+^IgD^+^IgM^+^ B cells can suppress osteoclast-mediated bone resorption and limit metastatic colonization, revealing a context-dependent protective role in skeletal homing (160). At the primary site, early B-cell presence in TNBC can induce IL-1β secretion, activating NF-κB signaling and enhancing matrix metalloproteinase activity, invasion, and migration (88).

### B cells and therapeutic resistance

B cells contribute to drug resistance in advanced breast cancer. FOS^+^ B cells, identified across multiple cancer types including breast cancer, drive differentiation into immunosuppressive plasma cells via AP-1–mediated BLIMP-1 expression and are linked to poor responses to immunotherapy (161). In hepatocellular carcinoma, BAP31 stabilizes SERPINE2 to promote tumor cell proliferation, suggesting analogous mechanisms may operate in breast cancer (162). Additionally, evidence from broader pan-cancer models demonstrates that B-cell-derived GABA promotes the differentiation of monocytes into anti-inflammatory macrophages that secrete IL-10, suppressing CD8^+^ T-cell cytotoxicity (163). Extrapolating this mechanism to breast cancer suggests it could potentially limit chemotherapy-induced immunogenic cell death.

### Tertiary lymphoid structures as organizational and functional hubs

The spatial organization of B cells into tertiary lymphoid structures (TLSs) fundamentally influences their developmental trajectory and functional output (164). These structures serve as the engine of an ‘intratumoral immunity cycle,’ where B cells are recruited, activated, and undergo local differentiation into memory and antibody-secreting plasma cells directly within the tumor bed (165). In breast cancer, TLSs range from immature lymphoid aggregates to mature structures containing defined B-cell follicles, active GCs with CD21^+^ follicular dendritic cells, Tfh-cell zones, and high endothelial venules (166, 167). Within mature TLSs, B cells undergo somatic hypermutation and class switching, generating high-affinity, tumor-specific plasma cells that migrate into the tumor bed along fibroblast tracks to secrete anti-tumor antibodies (168, 169). CXCL13-expressing Tfh cells are closely associated with TLS formation, and the CXCL13–CXCR5 axis is essential for B-cell organization (170).

However, not all TLSs are functionally equivalent. Some TLSs characterized by aberrant tryptophan metabolism, insufficient expression of the GC transcription factor BCL6, and upregulation of immunosuppressive enzymes such as IDO1 and TDO2 fail to support productive GC reactions and are instead linked to immune evasion (171). The phenotypic and metabolic state of B cells within these structures therefore determines whether TLS formation augments anti-tumor immunity or inadvertently reinforces tolerance (97, 172, 173). In breast cancer, the presence of somatically mutated immunoglobulin genes and antibody-producing plasma cells within TLSs signifies potentially localized evolution of the B-cell response, yet the maturational stage of these structures must be considered when interpreting their clinical significance (21, 174).

### Immune checkpoint expression and B-cell intrinsic regulation

B cells in breast cancer express a repertoire of immune checkpoint molecules that modulate their function. PD-1^+^ and PD-L1^+^ TIL-Bs have been identified in human breast cancer, suggesting that B cells are direct targets of PD-1/PD-L1 blockade (175, 176). PD-1 is distinctively expressed in Tfh cells within GCs, driving TLS formation and long-lived plasma cell generation through interactions with PD-L1/PD-L2 on B cells (177). Memory B cells upregulate PD-L2 compared with naive B cells, and high PD-L2 expression in GCs of tumor-draining lymph nodes may be associated with improved long-term prognosis (178). These observations indicate that immune checkpoint pathways operate not only on T cells but also intrinsically within the B-cell lineage to regulate humoral anti-tumor immunity.

### Chemotherapy-induced B-cell reprogramming

Systemic therapy profoundly alters B-cell function in breast cancer (179, 180). Neoadjuvant chemotherapy induces a phenotypic transition from IL-10-producing suppressive subsets toward ICOSL^+^ immunostimulatory B cells, and the magnitude of this shift correlates with treatment efficacy and long-term survival (112). Complement signals determine the directional effects of B cells in chemotherapy-induced immunity: in responder tumors, B cells upregulate ICOSL and CR2 while downregulating IL-10, promoting anti-tumor T-cell immunity. These chemotherapy-induced B-cell alterations underscore that the functional impact of TIL-Bs is temporally dynamic and therapeutically modifiable (112).

## Potential treatment based on B-cells

**Figure 2:**
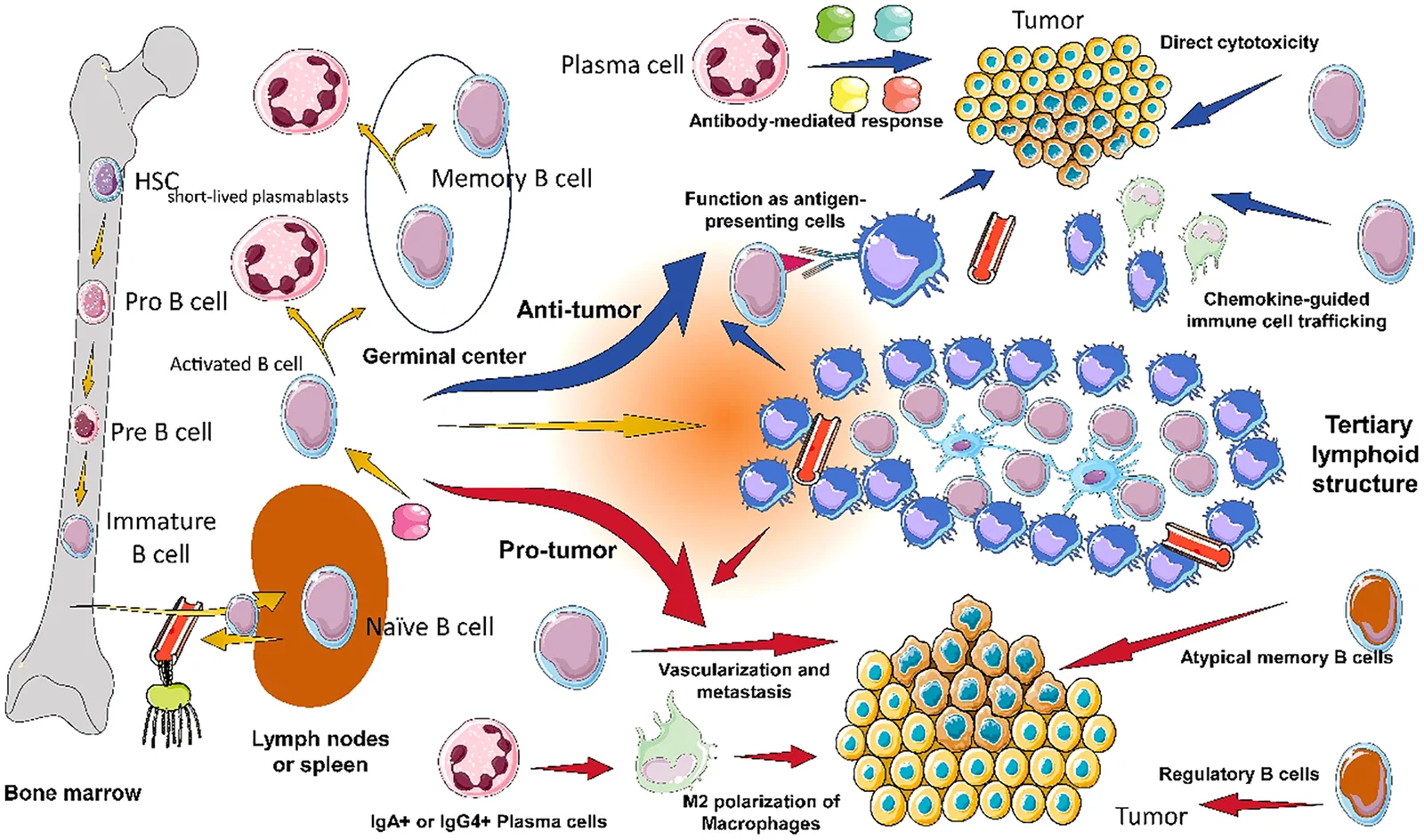
The B-cell landscape in breast cancer: developmental ontogeny and dualistic function. The left panel depicts systemic B-cell development. The right panel illustrates their opposing roles within the tumor microenvironment. The anti-tumor axis (blue) features antibody-mediated cytotoxicity, antigen presentation, and immune organization within tertiary lymphoid structures (TLSs). Conversely, the pro-tumor axis (red) highlights immunosuppressive mechanisms driven by regulatory B cells (Bregs) and atypical subsets.

The dualistic nature of tumor-infiltrating B cells in breast cancer presents a complex yet promising therapeutic opportunity (**Figure 2**). Unlike T-cell-centric immunotherapies that have achieved broad clinical success, B-cell-directed interventions remain largely preclinical or exploratory (42). However, the expanding recognition that B-cell function outweighs simple quantity in determining clinical outcome provides a rational framework for translating B-cell biology into precision therapeutics. Below, we outline emerging strategies to harness, reprogram, or selectively deplete distinct B-cell compartments, with emphasis on their integration into existing breast cancer treatment paradigms.

### Stratifying functional states: when to deplete and when to enhance

A central prerequisite for B-cell-targeted therapy is the accurate stratification of patients according to the dominant functional polarity of their intratumoral B-cell infiltrate. In tumors enriched in regulatory B cells (Bregs), atypical memory (AtM) B cells, or IgA-secreting plasma cells that drive immune evasion, therapeutic depletion may be advantageous (72, 147). Conversely, in tumors harboring mature tertiary lymphoid structures (TLSs), clonally expanded IgG1/IgG3-switched plasma cells, or CD200^+^ naive B cells associated with productive germinal center (GC) reactions, strategies that augment humoral immunity are warranted (181).

Anti-CD20 monoclonal antibodies such as rituximab, established in B-cell malignancies and autoimmune diseases, have been proposed as a means to eliminate suppressive B-cell subsets (182). Yet this approach carries a critical caveat: indiscriminate B-cell depletion risks eradicating anti-tumor effector populations while potentially enriching CD20-low Bregs that resist rituximab-mediated cytotoxicity and further entrench immunosuppression (183, 184). Recent pan-cancer analyses indicate that FOS^+^ B cells drive differentiation into immunosuppressive plasma cells via AP-1–mediated BLIMP-1 expression, linking specific transcriptional programs to therapeutic resistance (161). These observations underscore the need for subset-specific targeting rather than pan–B-cell ablation. Emerging chemical modulators, including STAT3 and Bruton’s tyrosine kinase (BTK) inhibitors, as well as CpG oligodeoxynucleotides, have demonstrated a preclinical capacity to suppress Breg expansion and attenuate IL-10 and TGF-β secretion in various hematological and solid tumor models. These findings provide a strong rationale for testing whether these targeted agents can mitigate Treg induction and restore cytotoxic T-cell function in breast cancer without wholesale B-cell elimination (185, 186).

### Immune checkpoint modulation and B-cell-intrinsic targeting

Immune checkpoint inhibitors (ICIs) have revolutionized breast cancer care, particularly in triple-negative breast cancer (TNBC). Although conventionally viewed through a T-cell lens, PD-1 and PD-L1 are expressed on subsets of TIL-Bs, and PD-1/PD-L1 blockade directly modulates B-cell activation, GC dynamics, and antibody production. In breast cancer, high baseline B-cell abundance and TLS maturity predict favorable response to neoadjuvant pembrolizumab plus chemotherapy. Integrative single-cell and spatial transcriptomic atlases have identified CD200^+^ naive B cells as a population tightly linked to TLS formation, improved clinical outcomes, and enhanced anti–PD-1 efficacy; functional validation in murine models confirms that this subset is essential for optimal anti-tumor immunity and ICI response. Conversely, proliferative CD19^+^Ki67^+^ B cells, which enhance TNBC organoid invasion and brain metastatic potential through NAMPT/ITGA5/ITGB1 signaling, correlate with poor overall survival and may define an ICI-resistant microenvironment.

### Metabolic and microenvironmental reprogramming

The functional trajectory of TIL-Bs is dynamically governed by metabolic cues within the tumor microenvironment (TME), offering a therapeutic window to redirect B-cell fate. Tryptophan metabolism via TDO2 and IDO1 generates kynurenine, fostering an immunosuppressive milieu that impairs TLS maturation and suppresses B-cell class switching (100, 187). Preclinical data demonstrate that restricting dietary tryptophan increases the proportion of mature, functionally competent TLSs and, when combined with PD-1 inhibitors, significantly reduces tumor burden (100, 188). Similarly, glutamine enrichment biases naive B-cell differentiation toward exhausted AtM phenotypes at the expense of productive GC responses; targeting glutaminase isoforms or altering amino acid availability may therefore preserve anti-tumor humoral immunity (189).

### Agonistic pathways, cytokines, and vaccine platforms

Strategies to actively boost anti-tumor B-cell responses are advancing through multiple modalities. Agonistic anti-CD40 antibodies activate B cells and dendritic cells, enhance antigen presentation, and reprogram macrophages toward pro-inflammatory states (190). In preclinical cancer models, localized delivery of anti-CD40 agonists in combination with anti–PD-1 and chemotherapy slows tumor growth while mitigating systemic toxicity, highlighting the promise of implantable or intratumoral delivery systems for B-cell-activating agents (190, 191).

Cytokine networks that orchestrate B-cell trafficking and differentiation represent additional targets. The CXCL13–CXCR5 axis is essential for B-cell organization and TLS formation; augmenting this chemokine circuit, either through engineered stromal cells or direct administration, may promote ectopic lymphoid neogenesis (192, 193). Similarly, BAFF and APRIL, produced by cancer-associated fibroblasts, sustain B-cell survival and plasma cell differentiation, and their therapeutic modulation could enhance local antibody production (194). Interleukin-21 (IL-21), a critical driver of T follicular helper (Tfh) cell and B-cell collaboration, can be leveraged to amplify GC reactions and boost IgG-switched, high-affinity humoral responses (177, 195, 196).

## Challenges and perspectives

Despite mounting evidence that tumor-infiltrating B cells (TIL-Bs) profoundly shape breast cancer biology, several major challenges must be overcome before B-cell-centric biomarkers and therapeutics can enter routine clinical practice. Foremost is the need to resolve functional heterogeneity at single-cell and spatial resolution. B cells operate as double-edged swords: clonally expanded, class-switched IgG1/IgG3 plasma cells and CD200^+^ naive B cells within mature tertiary lymphoid structures (TLSs) orchestrate potent anti-tumor immunity, whereas regulatory B cells (Bregs), atypical memory B cells, and IgA-secreting plasma cells actively suppress effector responses and promote metastasis. A major limitation of earlier clinical studies is their reliance solely on pan-B-cell immunohistochemistry, such as basic CD20 staining, which obscures these opposing functional programs and risks the misstratification of patients. Furthermore, there remains a significant lack of consensus surrounding the precise phenotypic definition of specialized subsets, particularly regulatory B cells and atypical memory or double-negative B-cell populations, which currently complicates cross-study comparisons. We urgently need integrative frameworks that simultaneously capture B-cell lineage, differentiation state, antibody isotype, metabolic fitness, and spatial architecture to distinguish protective from pathogenic infiltrates.

Additionally, it is important to acknowledge that the vast majority of current evidence regarding the prognostic and predictive value of B cells is derived from the more highly immunogenic breast cancer subtypes, namely triple-negative and HER2-positive disease. The role, spatial organization, and clinical utility of B cells in estrogen receptor (ER)-positive breast cancer remain comparatively under-explored and warrant dedicated future investigation. As the field moves toward clinical implementation, proposed B-cell and TLS metrics must not be viewed in isolation. Instead, future diagnostic frameworks should define how these novel humoral biomarkers can seamlessly complement existing standard-of-care evaluations, such as routine stromal TIL assessment and PD-L1 scoring, to generate a truly comprehensive immune profile for each patient.

Another critical hurdle is the standardization of B-cell metrics within diagnostic workflows. While TLS density, plasma-cell localization, and B-cell receptor repertoire skewing have shown prognostic and predictive value, particularly in triple-negative and HER2-positive breast cancer, assays remain fragmented across institutions. The field must establish consensus guidelines for multiplex immunohistochemistry, spatial transcriptomics, and computational deconvolution of bulk RNA sequencing, ensuring reproducible quantification of TLS maturity, germinal-center activity, and peritumoral versus intratumoral B-cell distribution. Moreover, dynamic peripheral B-cell signatures during neoadjuvant chemotherapy or immune-checkpoint blockade offer minimally invasive monitoring opportunities, yet prospective validation in multi-center trials is still lacking.

Therapeutically, the dualistic nature of B cells demands a precision paradigm fundamentally different from indiscriminate depletion. Although anti-CD20 antibodies such as rituximab eliminate suppressive subsets, they concomitantly eradicate effector populations and may enrich rituximab-resistant Bregs. Moving forward, subset-specific strategies, targeting STAT3 or Bruton’s tyrosine kinase to curtail Breg expansion, blocking PD-1/PD-L1 axes intrinsic to B-cell germinal-center dynamics, or leveraging agonistic anti-CD40 to potentiate antigen presentation, must be guided by prespecified biomarker profiles. Equally promising is metabolic reprogramming: restricting tryptophan availability or inhibiting IDO1/TDO2 can rescue TLS maturation and class-switching, while modulating glutamine flux may prevent exhaustion of atypical memory B cells. These approaches will likely achieve maximal benefit when rationally combined with existing T-cell-directed immunotherapies and chemotherapy, capitalizing on the chemotherapy-induced shift from IL-10^+^ suppressive B cells toward ICOSL^+^ immunostimulatory phenotypes.

Despite the comprehensive synthesis provided in this Review, several limitations must be acknowledged. First, much of the clinical evidence regarding TIL-Bs and TLSs is derived from retrospective cohorts utilizing highly variable detection methods—ranging from single-marker immunohistochemistry to advanced spatial transcriptomics and single-cell RNA sequencing. This methodological heterogeneity complicates direct cross-study comparisons. Second, the lack of universally accepted phenotypic markers for critical functional subsets introduces inherent variability in how these populations are defined and quantified across the cited literature. Third, while robust, many of the mechanistic insights regarding B-cell and tumor crosstalk rely on preclinical murine models, which may not fully recapitulate the complex and dynamic immunological landscape of human breast cancer. Finally, as with any narrative review, there is a potential for publication bias, wherein studies reporting positive prognostic associations may be overrepresented. Future prospective, multi-center clinical trials utilizing standardized, multiplexed profiling are essential to validate the functional paradigms and prognostic metrics discussed herein.

Looking ahead, the convergence of single-cell multi-omics, spatial transcriptomics, and artificial intelligence will enable the construction of high-resolution B-cell atlases across breast cancer subtypes and treatment stages. Such integrative maps should decode the crosstalk between B cells, T follicular helper cells, fibroblasts, and myeloid compartments, revealing context-dependent rules that govern humoral anti-tumor immunity. Ultimately, incorporating B-cell function—rather than mere quantity—into next-generation pathological reporting and adaptive trial designs represents an essential frontier. By transcending the traditional T-cell-centric view and embracing the full complexity of adaptive immunity, we can unlock novel therapeutic vulnerabilities and meaningfully improve outcomes for patients with breast cancer.
